# Electrostatic Embedding of Machine Learning Potentials

**DOI:** 10.1021/acs.jctc.2c00914

**Published:** 2023-02-23

**Authors:** Kirill Zinovjev

**Affiliations:** Departament de Química Física, Universitat de València, 46100 Burjassot, Spain

## Abstract

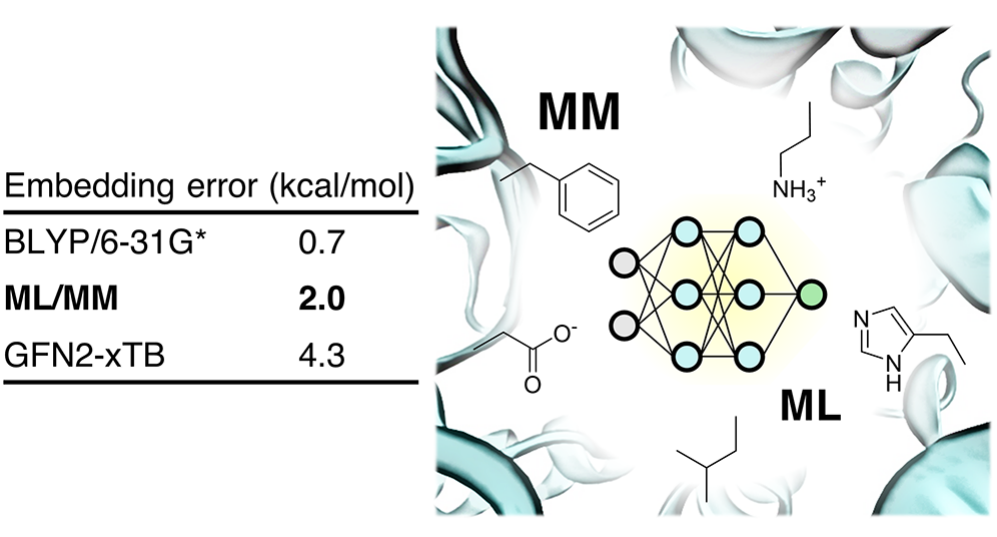

This work presents a variant of an electrostatic embedding scheme that allows the embedding of arbitrary machine learned potentials trained on molecular systems *in vacuo*. The scheme is based on physically motivated models of electronic density and polarizability, resulting in a generic model without relying on an exhaustive training set. The scheme only requires *in vacuo* single point QM calculations to provide training densities and molecular dipolar polarizabilities. As an example, the scheme is applied to create an embedding model for the QM7 data set using Gaussian Process Regression with only 445 reference atomic environments. The model was tested on the SARS-CoV-2 protease complex with PF-00835231, resulting in a predicted embedding energy RMSE of 2 kcal/mol, compared to explicit DFT/MM calculations.

## 1 Introduction

In the recent years, advances in Machine Learning^1−4^ and representations of molecular systems^5^ brought an explosion of Machine Learned (ML) potentials (or force fields), covering a broad range of systems and problems. In principle, ML potentials can provide energies and forces at arbitrarily high precision, while being orders of magnitude cheaper than DFT and *ab initio* calculations. However, the computational cost of ML potentials is much larger than the cost of molecular mechanics (MM) force fields.^6^ That makes their application to long scale MD simulations of systems consisting of ≈10^5^ or more atoms problematic. On the other hand, one often faces problems where a higher precision force field is required for just a small region or subsystem, while for the rest of the system, the precision of the classical force fields is satisfactory. For example, simulations of enzymatic catalysis rely on hybrid “QM/MM” calculations, with the active site of the enzyme (usually ≈100 atoms) being described by some QM method, while the rest of the system is treated at the MM level.^7,8^ This allows explicitly treating the electronic rearrangements at the desired QM level of theory while including the entire enzyme with the bulk water in the simulation system, keeping it realistic.

In state-of-the-art QM/MM methods, the interaction between QM and MM regions is usually treated by means of electrostatic embedding. The MM part is represented by a mesh of MM point charges surrounding the QM part, while the dispersion and repulsion interactions are treated at the MM level. The MM point charges are taken into account explicitly in the QM calculation, resulting in a polarized wave function or electronic density. The polarized density in turn interacts with the point charges, resulting in the total QM/MM energy expression.

The performance of QM/MM schemes is limited by the cost of the QM method used. Therefore, one could still obtain huge savings of computer time by employing a much cheaper ML model instead. However, the vast majority of ML potentials is trained to reproduce the energies of molecular systems as a whole, without considering the response to external electric fields. This makes it impossible to combine ML with MM in an electrostatic embedding scheme, unless the architecture of the ML model is modified to include the MM environment.

Very recently, several approaches have been proposed to tackle this problem. In the DPRc model by Zeng et al.^9^ a Δ-learning^10^ approach is proposed to correct QM/MM potentials to a higher level of theory. This is done by introducing a correction to the interaction energies that smoothly vanishes for MM atoms farther from the QM region. The environment is provided to the NN as positions and atom types of MM atoms. The method was recently applied to estimate free energy barriers and kinetic isotope effects in RNA cleavage reactions.^11^

An example of a more general approach aimed at predicting a range of response properties is FieldSchNet, proposed by Gastegger et al.^12^ In FieldSchNet, the description of the environment (such as the electric field caused by the MM point charges on each QM atom) is incorporated as an additional input in the NN architecture together with a physically motivated transformation (such as a dipole-field interaction tensor) added as an additional layer. The same philosophy but with a different network architecture was employed by Pan et al.,^13^ with the MM environment being represented by the generated electrostatic potential and field on QM atoms.

A step beyond reproducing a QM/MM potential is the BuRNN approach by Lier at el.,^14^ which also aims at reducing the artifacts at the QM/MM boundary. These are particularly pronounced when the interface is crossed by a covalent bond. In BuRNN, a buffer region is introduced where the interactions with QM and MM subsystems are treated at QM and MM levels, respectively.

In all aforementioned cases, the response to the MM environment is taken into account during the development of the ML potential. This contrasts the QM methods that “just work” with electrostatic embedding. Moreover, practical implementation of some of the mentioned schemes would imply substantial changes to the state-of-the-art QM/MM MD schemes, requiring, for example, extra information about the embedding (such as MM atom types^9,11^) or by introducing additional partitionings of the total system.^14^ The purpose of this study is to develop a modified electrostatic embedding scheme that would allow taking an existing ML force field (trained to reproduce energies *in vacuo*) and combining it with an arbitrary MM environment, resulting in an “ML/MM” potential.^12^ Moreover, such a scheme should only require the same representation of the MM subsystem that is currently available to QM engines in state-of-the-art QM/MM codes, that is the positions and charges of the MM atoms. This would allow construction of the full ML/MM potential by just replacing a QM engine by the embedding scheme and an *in vacuo* energy function, requiring minimal changes to well tested codes.

The feasibility of such a scheme follows from the fact that intermolecular interactions, in general, have relatively simple functional dependence on nature and geometry of the interacting species as was shown, for example in the IPML model by Bereau et al.^15^ The QM/MM interaction energy can be seen as a special case of intermolecular interaction, where some of the species are reduced to nonpolarizable point electronic density. In this case, the total interaction energy between the QM and MM subsystems reduces to the electrostatic interaction and polarizability components, which, as shown in Bereau et al. and also in this work, can be described using simple, physically motivated models with only a few free parameters.

The paper is outlined as follows. First, we show how standard electrostatic embedding can be reformulated to explicitly depend on the *in vacuo* energy of the QM part, thus allowing substitution of the corresponding term with an ML energy function. Then, a physically motivated model for the QM/MM interaction term is proposed, and the necessary atomic properties as well as free model parameters are introduced. The scheme is then used to create a generic embedding scheme applicable to ground-state geometries of neutral compounds containing H, C, N, O, and S elements by training it on the QM7 data set. Finally, the scheme is tested by predicting QM/MM interaction energies in the SARS-CoV-2 main protease complex with the PF-00835231 inhibitor. The paper is concluded by discussing advantages, limitations, and possible future improvements of the proposed embedding scheme.

## 2 Theory and Method

### 2.1 Decoupled Embedding

We start with the total energy of a QM/MM system treated within an electostatic embedding scheme, which can be written as the sum of four terms^7^
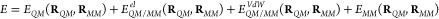
1where the first term is the energy of the QM subsystem polarized by the MM point charges, the second term is the interaction energy between the polarized QM subsystem and the point charges, the third term is the VdW interaction energy between QM and MM parts, and the fourth term is the MM energy. The last two terms are treated at the MM level of theory and therefore are trivial to calculate. For conciseness, **R**_*MM*_ denotes dependence on both the positions of MM atoms **r** and their point charges *q*. Due to the dependence of the QM term on **R**_*MM*_, it cannot be replaced by an ML model trained on *in vacuo* energies, thus making the standard electrostatic embedding unsuitable. To overcome this issue, we split the QM term into two components:
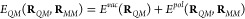
2Now, the first term is the SCF energy of the QM subsystem in the absence of MM part and therefore can be replaced by some ML model trained on energies *in vacuo*. The second term contains the polarization energy cost required to distort the electronic density in response to the external potential.

Now we can rewrite the total energy expression as
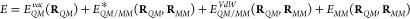
3where
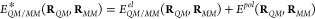
4On the r.h.s. of eq 3, we have “decoupled” the energy of the QM system *in vacuo* and the “embedding” term that includes all the effects of the MM environment and has to be predicted in the modified embedding scheme. The next section discusses how this term can be obtained from a simple physically motivated model.

### 2.2 The Embedding Term

The electrostatic QM/MM term in (4) is the interaction energy between the polarized QM part and the MM point charges, which can be obtained from the electrostatic potential corresponding to the polarized density
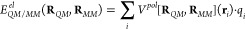
5where **r**_*i*_ and *q*_*i*_ are positions and charges of the MM point charges, respectively. Here and below *V*[**X**](**r**) denotes a potential defined by some arguments **X** and estimated at point **r**. *V*^*pol*^ is the electrostatic potential of the polarized QM part which can be further decomposed
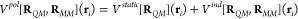
6where *V*^*static*^ is the electrostatic potential of the **nonpolarized** QM part, and *V*^*ind*^ is the “**induced** potential” due to the change in the charge distribution of the QM subsystem caused by the presence of the MM environment. Combining (4), (5), and (6), the embedding term can be written as
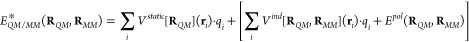
7To calculate the first term on the r.h.s. of (7), one only requires *V*^*static*^, which is the electrostatic potential of the QM part in vacuo. Modeling *V*^*static*^ is a well-known problem that arises, for example, when developing partial charge schemes for classical force fields. Therefore, in principle, any existing approach that aims to represent the electostatic potentials can be employed to calculate this term. For the purpose of this work, we use a *V*^*static*^ model based on a simple approximation of the *in vacuo* electronic density that is described in section 2.3.

The second part on the r.h.s. in (7) corresponds to the response of the QM part to the presence of the MM point charges. To calculate it, one must both obtain the changes in the electrostatic potential due to polarization of the QM part (*V*^*ind*^), as well as quantify the corresponding polarization energy cost *E*^*pol*^. Both terms can be obtained from the Thole model^16^ which relies on damped atomic polarizabilities and is described in section 2.5.

### 2.3 Static Density Model

In principle, any approach to construct the electrostatic potential could be employed to calculate the static term in (7). The particular choice would depend on the desired properties of the resulting model, such as its generality and computational cost. Here we construct the approximation of the electronic density based on minimal basis iterative stockholder^17^ (MBIS) partitioning because of the consistency of MBIS charges, simplicity of the resulting expression for electostatic potential, and the fact that an analytic expression is provided for atomic volumes, which are required for the induction model (section 2.5). In MBIS, the total molecular electronic density ρ^*MBIS*^ is approximated as a sum of atomic contributions ρ_*i*_^*MBIS*^:
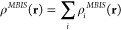
8Here and below the summation goes over the atoms in the QM subsystem. Each atomic contribution consists of two charge densities: the core charge with *N*_*i*_^*core*^ electrons localized at the nucleus representing the sum of the nuclear charge and that of the core electrons and the valence charge with *N*_*i*_^*val*^ electrons representing the outer electronic shell and approximated by a Slater function with width *s* centered at the nucleus
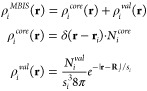
9where **R**_*i*_ is the position of the nucleus *i*. This charge distribution results in the following expression for the electrostatic potential^18^
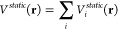
10
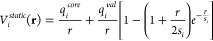
11where *r* = |**r** – **R**_*i*_|, and *q*_*i*_^*core*^ and *q*_*i*_^*val*^ are the charges of core and valence charge distributions, respectively (*q* = −*N*).

### 2.4 Charge Equilibration

An advantage of the charge density model described in section 2.3 is that it provides a reasonable description of *V*^*static*^ without the need to include higher order atomic multipoles (dipoles, quadrupoles, etc.). However, the quality of the resulting *V*^*static*^ does strongly depend on the atomic charges, which are known to have nonlocal character: a strongly electronegative chemical group multiple bonds away may notably impact the charge on a given atom.^4,19^ To handle this issue, we employ the charge equilibration scheme (QEq)^19,20^ to predict the total atomic charges (*q* = *q*^*core*^ + *q*^*val*^) based on atomic electronegativities. In QEq, the atomic charges are treated as Gaussian charge distributions
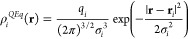
12which are obtained by minimizing the following expression
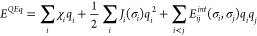
13where the first term corresponds to the interaction between the charges and corresponding atomic chemical potentials or electronegativities (χ), the second term is the energy cost of creating the partial charges, and the third term is the electrostatic interaction energies for each charge pair. *J*_*i*_ is the chemical hardness and is calculated as self-energy of a normal charge distribution^21^
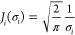
14and *E*_*ij*_^*int*^ is the interaction energy between two normal densities
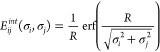
15with *R* being the distance between the nuclei. Differentiating (13) w.r.t. *q* and adding a constraint on the total charge of the molecule give the following set of linear equations for the charges
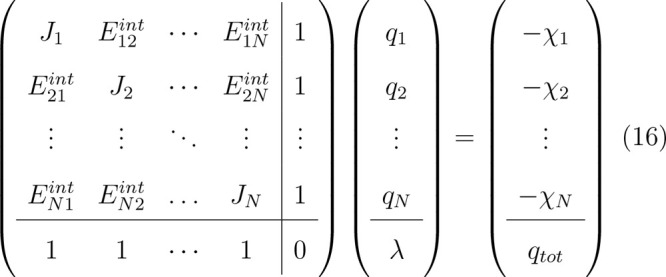
16where λ is a Lagrange multiplier that constrains the sum of charges to *q*_*tot*_.

Apart from electronegativities, QEq requires the widths σ of Gaussian charge densities to be provided. Here we assume σ to be proportional to the Slater valence shell width from MBIS partitioning (eq 9)
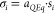
17where *a*_*QEq*_ is the universal scaling factor, independent of the nature of the atom, and a free parameter in the model. Finally, since core electronic shells are not sensitive to long-range effects, *q*_*core*_ can be modeled independently of QEq Then, the valence charge can be obtained from the QEq charges as *q*_*val*_ = *q* – *q*_*core*_, therefore including all the long-range contributions due to QEq.

### 2.5 Induction Model

To model the second term in (7), which corresponds to the response of the QM subsystem to the MM environment, we use the atomic polarizabilities model with Thole damping.^16^ Each atom is treated as a polarizable center with isotropic polarizability α_*i*_ where the values of the atomic dipoles induced by the external field are obtained by minimizing the total energy of the system:
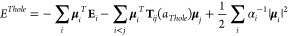
18The first term on the r.h.s. of (18) is the interaction energy between the induced dipoles **μ**_*i*_ and the external fields **E**_*i*_ formed by the MM point charges on each atom. The second term is the interaction between the induced dipoles themselves. This term is damped modifying the dipole–dipole interaction tensor **T** using the cubic exponential Thole damping^16^
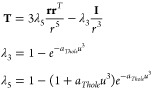
19where **r** is the interatomic vector,  is the reduced interatomic distance scaled by atomic polarizabilities, and *a*_*Thole*_ is the universal damping factor. The third term in (18) is the energy cost required to create the induced dipoles and is used as an estimate of *E*^*pol*^ in eq 7.

Differentiating (18) w.r.t. **μ** gives a system of 3N linear equations for **μ** components
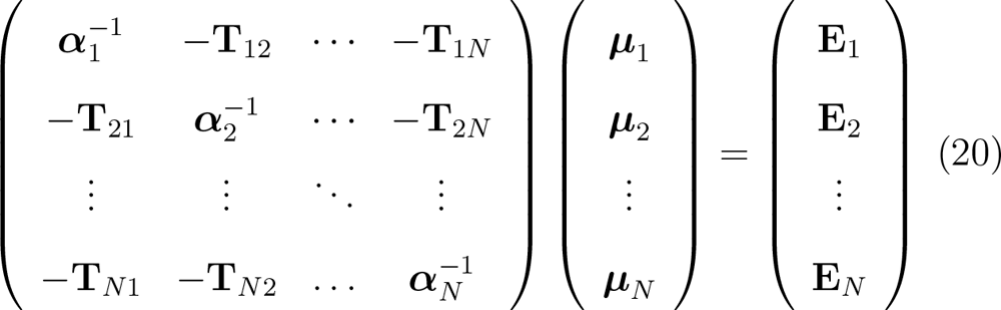
20where **E** are electric field vectors generated at nuclei by MM point charges, and **α**^–1^ = α^–1^**I** is a 3 × 3 diagonal matrix of α^–1^.

The induced electrostatic potential *V*^*ind*^ now can be computed as the total potential generated by the induced atomic dipoles:
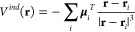
21A commonly used approximation to obtain atomic polarizabilities α is to assume them to be proportional to the atomic volume:^22^
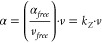
22Here *k*_*Z*_ is the polarizability/volume ratio of free atoms (different for each chemical element) treated as free parameters. To obtain the atomic volumes, we again take advantage of MBIS partitioning employed in the static density model (section 2.3) and calculate the volume as the third radial moment of the atomic Slater density:
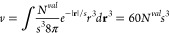
23Since *N*^*val*^ and *s* already have to be predicted for the static part of the embedding model, no extra properties have to be learned.

## 3 Implementation

As a practical example, below we will build an embedding model for ground state neutral compounds containing H, C, N, O, and S elements. As discussed above, the scheme relies heavily on physically motivated models and therefore can be expected to require little training data and be able to make predictions outside the training domain, since the functional capacity required to describe the embedding energies is already to a large extent encoded in MBIS, QEq, and Thole models. To emphasize that, we build a Gaussian Process Regression (GPR)^3,23^ model of atomic properties required by the scheme using only a handful of reference atomic environments. We also intentionally consider only environments with a short distance cutoff, to show the ability of QEq and Thole models to handle long-range effects without explicitly learning them. Finally, despite training the model exclusively on *in vacuo* QM data for small molecules, we apply it to a large QM system with an explicit MM environment to show that the model performs well beyond its training domain.

### 3.1 Data Set and Reference QM Calculations

Training data was generated based on the QM7 data set,^24,25^ consisting of 7165 molecules with up to 7 heavy atoms (C, N, O, and S, in addition to H). For each molecule, the density and molecular dipolar polarizability were obtained at the B3LYP/cc-PVTZ level of theory without reoptimizing the structures. All calculations were performed with ORCA 5.0.3.^26^ 4 (congeneric) molecules with highly distorted geometries were excluded from the data set (see SI, section S1 for details). 80% of the full data set (5729 molecules) were randomly chosen as a training set, while the remaining 20% (1432 molecules) were used as a test set.

The atomic environments were represented using SOAP^3,27^ feature vectors calculated with the librascal package (https://lab-cosmo.github.io/librascal/). The distance cutoff was set to *r*_*cut*_ = 3 Å, and angular and radial channels were limited to *l*_*max*_ = 4 and *r*_*max*_ = 4, respectively. The ζ = 2 polynomial SOAP kernel^3^ was used, and the representative (basis) set of 445 atoms was chosen from the training set using the Informative Vector Machine^23,28^ with a variance threshold of 0.05 (see SI, section S2 for details). The learning was performed on a single NVIDIA V100 (Volta) GPU using the JAX package (http://jax.readthedocs.io/).

### 3.2 Model Fitting

All the properties and parameters required by the embedding scheme and the learning approach are listed in Table 1.

**Table 1:** Model Parameters

property	number of parameters	learning approach
core charge (*q*^*core*^)	5 (1 per element)	average MBIS values over training set
valence width (*s*)	445 (1 per basis atom)	sparse GPR to MBIS values
electronegativity (χ)	445 (1 per basis atom)	least squares of predicted MBIS charges
width scaling factor (*a*_*QEq*_)	1	
polarizability/volume ratio (*k*_*Z*_)	5 (1 per element)	least squares of predicted molecular dipolar polarizability components
Thole damping factor (*a*_*Thole*_)	1	

The core charges are highly consistent across QM7 within each element, so the average values can be used directly, without building a prediction model. This limits the number of parameters needed to predict *q*^*core*^ to only 5 (1 for each element). The valence widths are obtained by applying a modified sparse GPR (see SI, section S3 for details) to the training set with 445 fitted valence width values corresponding to the representative atoms.

The atomic electronegativities required by the QEq model are not available directly from MBIS partitioning and therefore have to be fitted (together with *a*_*QEq*_) by minimizing the Mean Square Deviation (MSD) between the training set charges predicted by QEq (given specific values of χ) and the corresponding MBIS charges. Loss function minimization was performed using the Adam optimizer.^29^ Note that only 445 electronegativities of the reference basis atoms are learned, while the values for the training set used to predict *q* are then obtained with regular GPR.

Once all the parameters for the static component of the model are obtained, the atomic volumes can be predicted by eq 23, and the 6 parameters required by the induction model (5 *k*_*Z*_ and *a*_*Thole*_) can be fitted by minimizing the MSD between the molecular dipolar polarizability components obtained from the Thole model (see SI, section S4 for details) and the corresponding DFT values. The Jupyter notebook with the training protocol is available in the GitHub repository (https://github.com/emedio/embedding), and the training workflow is provided in the SI (section S11).

### 3.3 Training Results

**Figure 1:**
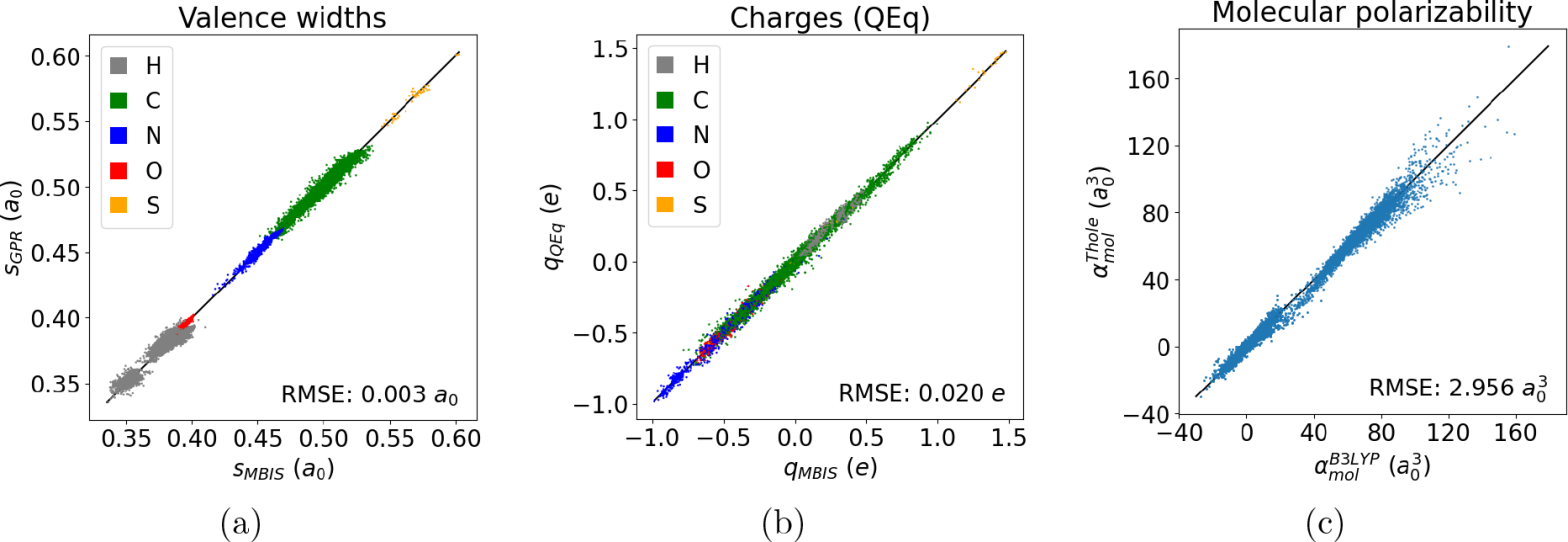
Predictions and RMSE for the test set (1432 molecules). a) valence width (*s*), obtained by sparse GPR fitting of reference values (*s*) to MBIS valence widths; b) atomic charges (*q*), obtained by least-squares fitting of reference electronegativities (χ) and *a*_*QEq*_ to MBIS values; c) molecular dipolar polarizability components, obtained by least-squares fitting of *a*_*Thole*_ and *k*_*Z*_ to DFT values.

The quality of fit is estimated by comparing the predicted values of *s*, *q*, and **α**_*mol*_ (molecular dipolar polarizability) to the reference DFT data (Figure 1). Although only 445 kernels were used for GPR, the prediction is reasonably good for all the properties learned, indicating that the dependence of the learned properties on the atomic environments is smooth and therefore can be captured with only a few observations. Also, as shown in Table 2, the errors for training and test sets are remarkably consistent. This indicates that the model is likely underfit and the errors might be lowered by increasing the number of free parameters in the model, e.g. by expanding the representative set of atoms. However, here the functional capacity of the model was limited on purpose, to emphasize the predictive power of the physically motivated energy expression used.

**Table 2:** Model Prediction Errors for Reference DFT Properties

	training set	test set
valence width, *s* (*a*_0_)	0.003	0.003
charge, *q* (*e*)	0.02	0.02
polarizability, α_*mol*_ (*a*_0_^3^)	2.98	2.96

For charges (*q*) and valence widths (*s*), the relative prediction errors are largest for hydrogen atoms (see SI, section S8). This can be explained by the fact that for more “crowded” chemical groups such as branched aliphatic chains, the partitioning of the density to the atomic contributions is not as well-defined as in case of e.g. the oxygen atom of a ketone group. This can be interpreted as the noise in the reference *q* and *s* values, which is actually undesirable to learn. This conclusion is supported by two observations. First, the consistency between training and test set errors mentioned above indicates that the model does not suffer any overfitting, so no possible noise in the training data was learned. Second, prediction of molecular dipolar polarizability components does not get improved when exact MBIS values of *q* and *s* are used to obtain atomic volumes, even though the volumes predicted by the model differ from the MBIS ones (see SI, section S5). That indicates that the information lost in *q* and *s* models is irrelevant to molecular polarizabilities and therefore is physically meaningless.

Remarkably, despite the simplicity of the model, the predicted components of dipolar polarizability tensors are in very good agreement with the QM data, with an RMSE of 3*a*_0_^3^ for the test set. This error is comparable to typical DFT polarizability errors relative to CCSD(T).^30^ As shown in the next section, this precision makes the “static” component of the embedding, independent of the MM part, the largest contribution to the prediction error.

### 3.4 Test Case: The SARS-CoV-2 Mpro Complex with PF-00835231

**Figure 2:**
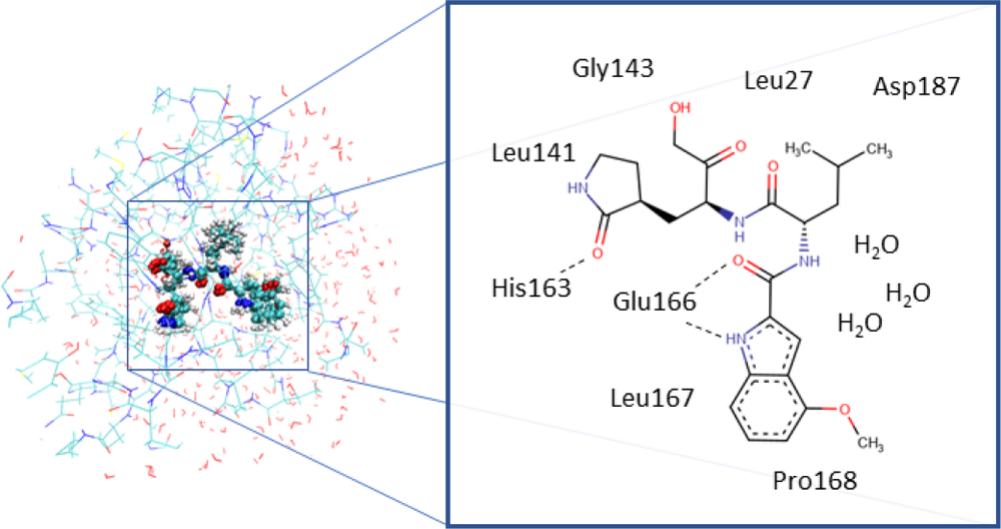
3D structure and schematic representation of the SARS-CoV-2 Mpro active site with PF-00835231.

The trained model was tested by calculating the embedding energies corresponding to the noncovalent complex formed by the PF-00835231 inhibitor and SARS-CoV-2 main protease, previously studied by our group (see Ramos et al.^31^ for details). This system serves as a good test case for an embedding model: the ligand has relatively rich chemistry, including polar, nonpolar, aromatic groups, and heterocycles. The MM environment includes charged, polar, and neutral groups, and the ligand is partially exposed to the bulk solvent (Figure 2). 100 evenly spaced snapshots from a 1 μs long MD trajectory were taken, and single point energies with and without the MM point charges (up to 12 Å from the closest QM atom) were obtained at the B3LYP/cc-PVTZ level of theory (same that was used to generate the training data set). The difference between the energies with (*E*_*QM*_(**R**_*QM*_, **R**_*MM*_)) and without (*E*_*QM*_(**R**_*QM*_)) MM point charges is the quantity that the embedding model aims to predict:
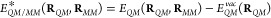
24To separately assess the quality of the static and induced components of the embedding energy, the interaction between the nonpolarized (*in vacuo*) QM system and the MM point charges was obtained by calculating the value of the electrostatic potential of the QM part at positions of MM atoms and evaluating the first term on the r.h.s. of (7) (except for semiempirical methods, see SI, section S6). Finally, to analyze the performance of MBIS density and QEq models, embedding energies were obtained by 1) replacing predicted charges by their MBIS values averaged over all 100 snapshots, 2) replacing predicted electronegativities by their averaged values derived from MBIS charges (see SI, section S7), and 3) using exact MBIS charges for each snapshot.

The prediction RMSE for the proposed “ML/MM” embedding model as well as for DFT calculations with smaller basis sets and for semiempirical methods is shown in Table 3. The large absolute RMSE values for different DFT methods (see SI, section S9) are most likely due to charge spillover, which is supported by significant systematic interaction energy differences between B3LYP/cc-PVTZ and B3LYP/cc-PVDZ. Therefore, the analysis was performed by removing the error of the mean, which would not affect MD simulations with a given method. Despite the model being trained only on *in vacuo* QM calculations for small molecules, it was able to predict embedding energies of a large ligand with an explicit MM environment with an RMSE of 2 kcal/mol. The error is large compared to cheaper DFT calculations but significantly lower than that of tested semiempirical methods. Moreover, the prediction error is dominated by the static component (RMSE = 1.9 kcal/mol), with the induced component having a significantly lower RMSE of 0.6 kcal/mol. The error of the proposed model is also lower than the one obtained when a different force field (OPLS-AA) is used to provide the MM charges, indicating that the classical force field becomes the error bottleneck once the quality of the QM Hamiltonian goes beyond the semiempirical methods.

**Table 3:** Embedding Energy Prediction RMSE (kcal/mol) and Execution Times (s) for the SARS-CoV-2 Mpro Complex with PF-00835231^a^

method	*E*_*Full*_	*E*_*Static*_	*E*_*Induced*_	*t*
B3LYP/cc-pVTZ+OPLS-AA	3.800	3.018	1.486	6802
PBE0/cc-pVTZ	0.214	0.217	0.022	6884
B3LYP/cc-pVDZ	0.639	0.504	0.307	2261
BLYP/6-31G*	0.655	0.539	0.326	429
ML/MM *q*_*MBIS*_	1.484	1.394	0.560	
ML/MM MBIS	1.490	1.398	0.549	
ML/MM ⟨χ_*MBIS*_⟩	1.760	1.644	0.561	
ML/MM ⟨*q*_*MBIS*_⟩	2.046	1.926	0.561	
ML/MM	2.046	1.941	0.567	0.05
GFN2-xTB	4.342			0.7
AM1	10.330			0.9
PM3	10.663			0.9

a The systematic error was removed by subtracting the mean in all cases (see text). B3LYP/cc-pVTZ+OPLS-AA - energies calculated with B3LYP/cc-pVTZ but using charges from the OPLS-AA force field instead of ffSB14; ML/MM - prediction based on the model trained in section 3.3; ML/MM MBIS - ML/MM with *q*_*core*_, *q*_*val*_, and *s* taken directly from MBIS partitioning; ML/MM *q*_*MBIS*_ - predicted charges replaced by their exact MBIS values; ML/MM ⟨χ_*MBIS*_⟩ - electronegativities replaced by their average MBIS values (see SI, section S7); ML/MM ⟨*q*_*MBIS*_⟩ - charges replaced by their average MBIS values. The execution times are single SCF time in the case of DFT and semiempirical methods and time to calculate the embedding energy in the case of ML/MM (using a single CPU core).

Replacing charges or electronegativities with their exact or average values reveals the following properties of the model. Replacing the charges predicted using QEq based on electronegativities learned from the QM7 data set by their average values (for each atom) from MBIS partitioning of the exact *in vacuo* DFT density leads to a model with the same precision. The role of QEq is to take into account both the long-range effects of charge redistribution and conformational dependence of the atomic charges. The error comes from the nature of the model - it is only trained on small molecules and uses very few reference environments. On the other hand, using average *q* values completely ignores the conformational dependence but in a sense provides “exact” treatment of long-range electronegativity effects. Both approximations result in roughly the same RMSE of the resulting models. The ⟨χ_*MBIS*_⟩ model goes one step further and does approximately account for conformational dependence of the charges (through QEq) but not for electronegativities (since average values are used). This brings the RMSE of *E*_*static*_ down to 1.6 kcal/mol. Directly using the charges from MBIS partitioning provides “exact” treatment of both long-range effects and conformational dependence and reduces the error further down to 1.4 kcal/mol. In this case, the values of *s* and *q*_*core*_ are still not taken from MBIS but provided by the model trained in section 3.3, supposedly introducing some error to the model. However, it turns out that using the exact values for all atomic properties (*q*_*core*_, *q*_*val*_, and *s*) directly from MBIS partitioning does not improve the prediction. That supports the assumption that any information lost by learning valence widths (see SI, section S10) is hindered by the noise in the reference data due the fact that the partitioning is often not well-defined (section 3.3). Ignoring this noise compensates the error introduced by the limited functional capacity of the model, resulting in overall the same prediction quality as for the “exact” MBIS density.

Another important conclusion is that using the exact MBIS density and, therefore, the “exact” atomic volumes does not provide any significant improvement to the induction component of the ML/MM model. This is partly explained by the fact that the volumes are calculated based on the valence charges, which include valence shell electrons of the atom, but not the core electrons or nuclei. That makes any charge variability much less pronounced in relative terms, resulting in less variability of the volumes and, therefore, the atomic polarizabilities. For example, the standard deviation of carbon atom charges in the system studied here is 0.34*e*, while the average *q*_*val*_ is −4.35*e*, resulting in relative volume s.d. of only ≈8%. On the other hand, atomic volumes also depend on the valence widths *s* (eq 23). As discussed above, *s* values provided by the model, while inevitably suffering from some learning error, might be free from the partitioning noise. This apparently results in overall the same “physical precision” of the *s* values compared to those provided by MBIS, explaining the same quality of the induction energy predictions.

## 4 Discussion

The results presented above show that a relatively simple model based on the proposed embedding scheme is able to provide embedding energies for a realistic QM/MM system with satisfactory precision, clearly beyond that of semiempirical methods, at a fraction of the computational cost. Even though the model is still inferior even to cheap DFT methods (such as BLYP/6-31G*), the result is surprisingly good for multiple reasons. First, the model was only trained on *in vacuo* calculations of small molecules (up to 7 heavy atoms) and then applied far beyond its training domain to a QM system consisting of 66 atoms (34 heavy ones) and with explicit MM point charges. Second, the model is generic and is expected to work for the chemical space covered by the QM7 data set, while requiring <1000 free parameters. Third, the induction component of the model, which is the one that explicitly depends on the configuration and charges of the MM environment, is predicted with high accuracy, within 0.6 kcal/mol of reference DFT values. These features of the model are due to the heavy reliance of the embedding scheme on physically motivated models, rather than on large training sets and ML models with high functional capacity that could accommodate the information content of rich training data. Therefore, this work is another example of how physics-based models with correct asymptotic behavior can dramatically reduce the amount of data and free parameters required to achieve satisfactory predictive power of a model.^15,32^ The Slater valence shells of MBIS partitioning approximately represent the typical decay of the electronic density and thus result in a meaningful description of the electrostatic potential, despite its simplicity. The QEq model captures the impact of all the chemical groups, even distant ones, on how the charge is distributed over the molecule. Finally, the Thole model and volume-based approximation of atomic polarizabilities relate the electronic structure to the response of the molecule to inhomogeneous external electric fields. These models capture a great share of functional capacity needed to reproduce the QM/MM interaction energies, leaving only a handful of free parameters to be explicitly fitted to the reference data. This is also emphasized by the fact that rather “miopic” feature vectors (*r*_*cut*_ = 3 Å) were sufficient to provide satisfactory results. It seems that the immediate chemical environment is enough to determine the atomic properties, while the impact of atoms beyond a 3 Å cutoff is well described by QEq and Thole models.

The obtained results are promising and suggest that the proposed embedding scheme might be a step forward toward a truly generic approach for the embedding of arbitrary machine learned potentials. However, the example model presented here and a single test case are not enough to draw any general conclusions. So far, the scheme has only been applied to train an embedding model for neutral molecules in their ground state geometries. It is yet to be shown, whether the same embedding scheme is adequate for charged species. Especially concerning is the treatment of anions due to higher polarizability and possible stability issues of reference *in vacuo* calculations needed to obtain density and polarizability data. On the other hand, since the scheme only requires reference QM data obtained from *in vacuo* simulations, one could train a model using large and diffuse basis sets without the risk of charge spill-out in the resulting ML/MM simulations, a known issue with negatively charged species.^33^ Regarding geometries, applicability to transition states is essential to describe chemical reactivity - the most common use case for QM/MM calculations. Changes in topology also would challenge the QEq model, if one wants to maintain an integer charge for each specie in the QM region both in reactant and product states. Finally, the model for now is only applicable to a handful of chemical elements. It is not clear whether more challenging systems, such as metals, would be adequately described with the simple embedding scheme proposed here. For instance, in its current state, no hyperpolarizability treatment is included in the scheme - induction is only described with point dipoles, and atomic α only depend on *in vacuo* density. In systems with metallic behavior, there is considerable charge flow in response to external fields,^34^ which means that calculation of atomic charges should be modified to account for this effect. Interestingly, once this is done, the coupling between QEq and the Thole model (α depend on volumes, which are determined by valence charges) would also result in response of dipolar polarizability to the external field. It is to be seen, whether this coupling helps to describe more complex response behaviors.

Given these caveats and assuming that the observed performance of the model is representative of other systems covered by QM7 chemical space, the trained model is already capable of improving the embedding in, for example, noncovalent binding free energy calculations of small molecules to biomolecular targets. Such calculations require extensive sampling of conformational space and therefore are performed at the MM level of theory. On the other hand, binding energies are mostly determined by the noncovalent interactions between the ligand and the host. Therefore, improved treatment of these interactions is desirable. The model trained here can be used together with some generic ML potential for small molecules (such as ANI-1^35^) to significantly improve the description of ligand-host interactions.

On the practical side, the only inputs required by the model (and the proposed embedding scheme in general) are the positions and element types of the QM atoms and the positions and charges of the MM atoms - exactly the information that is provided to the QM engine in QM/MM calculations. Therefore, the ML/MM embedding can be incorporated into existing QM/MM codes by “mimicking” the QM engine, without any substantial change to the code. That also allows the reuse of existing treatment of covalent bonds crossing the QM/MM boundary. In regular QM/MM, the link atoms are provided by the QM/MM code and treated by the QM engine as regular QM atoms. The same way, the ML/MM engine would only see additional atoms in the ML part and treat them as such, while all the technicalities would be taken care of by the QM/MM code.

Performance-wise, the proposed embedding model takes 50 ms on a single CPU core for a single energy estimate of a 66-atom inhibitor, being 4 orders of magnitude faster than the cheapest DFT method tested (BLYP/6-31G*) and at least 1 order of magnitude faster than semiempirical approaches. This efficiency is achieved due to reliance on physically motivated expressions, which results in a small number of free parameters and therefore simple model architecture. For instance, the model presented here uses only 445 reference atoms making the GPR predictions very cheap. If a deep learning model was used instead of GPR, one might expect very simple network architectures (with few neurons and layers) to be sufficient, also making the model highly efficient. Moreover, most of this computational cost is related to the calculation of the SOAP feature vectors and GPR predictions of valence widths and electronegativities. These tasks are trivially parallelizable and would get dramatic speed-up if performed on GPU, bringing the cost of the energy estimate close to that of the MM part (≈1 ms). Furthermore, when used alongside with an ML potential, in principle the same features could be used to predict both in vacuo and embedding energies, making the overhead of an embedding model negligible. It has to be noted that the QM subsystem chosen here is relatively small (66 atoms). For larger systems, the Thole model, which relies on solving a system of 3N linear equations, will likely become a bottleneck. However, the same problem arises in polarizable force fields, and iterative solutions exist to bring the computational cost down.^36,37^

The analysis performed by replacing the predictions of the ML/MM model by exact values of charges/electronegativities from MBIS partitioning of DFT densities (section 3.4) reveals the potential of the scheme for system-specific model training. For instance, using a single average value of electronegativity is analogous to using only a single basis atom for the corresponding GPR model. By including more system-specific observations, the prediction error could be brought down even more, potentially down to the value obtained with “exact” MBIS charges. The aim of the embedding scheme is to be coupled with some *in vacuo* ML potential. It is reasonable to assume that when such a potential is available, the reference QM data on the system of interest is abundant - the energy models generally require much more training data than what seems to be needed for the embedding. This data could be reused to train the embedding model, thus requiring no extra computational cost. The only condition is that the electronic density is stored and the dipolar polarizabilities are calculated upon training data set generation. However, the latter might not be needed, since the reference dipolar polarizabilities were only used to fit the free parameters of the Thole model (*a*_*Thole*_ and *k*_*Z*_). The values of these parameters obtained here are expected to be applicable to system-specific models as well, thus avoiding the need to calculate QM polarizabilities and retraining the induction model.

The decomposition of the predicted embedding energies into static and induced components (section 3.4) shows that the primary source of error (at least for the model presented here) is the static component, which relies on the prediction of the *in vacuo* electrostatic potential of the QM part. While some improvement can be achieved by using a system-specific model, one has to extend the scheme and go beyond the MBIS density approximation to achieve significant reduction of the prediction error. One way to do so is to introduce atomic multipoles (dipoles, cuadrupoles etc.), as was done, for example in IPML.^15^ In the current setup, this could be done efficiently reusing the spherical expansion coefficients used for the SOAP features to calculate the λ-SOAP ones^38^ which allow the learning of rotationally equivariant properties. Apart from that, the embedding scheme is, in principle, agnostic of how the electrostatic potential is predicted. So, if a better model is available, it could be straightforwardly employed instead of the one based on the MBIS density to obtain better predictions. Especially promising are the ML models that aim to directly predict the electronic density.^39−41^ In this case, not only the static component can be obtained but also the atomic volumes could be derived directly from the predicted density, completely bypassing the MBIS partitioning.

## 5 Conclusions

In this work, we introduced an alternative electrostatic embedding scheme that allows the embedding of arbitrary ML potentials in an MM environment. The embedding energy is calculated by separately predicting the static component, representing the interaction between the nonpolarized charge distribution of the system and the MM point charges, and the induction component, which incorporates the response of the system to the presence of MM environment. The proposed scheme relies on charge equilibration and MBIS partitioning to describe the electronic density and on the Thole model to treat the induction term. These models encode a large part of the functional capacity of the embedding energy, allowing training of models with relatively few free parameters. Moreover, the scheme only requires *in vacuo* reference QM data (densities and dipolar polarizabilities) for training.

The proposed scheme was used to train an embedding model based on DFT reference calculations of molecules in the QM7 data set. This resulted in a generic model, suitable to predict embedding energies of arbitrary neutral molecules consisting of H, C, N, O, and S elements in their ground state geometries. The model was tested by predicting QM/MM embedding energies of the noncovalent complex formed by the SARS-CoV-2 main protease with the PF-00835231 inhibitor. The predictions for embedding energies were more precise than those obtained from semiempirical Hamiltonians, while being ≈15 times faster.

The presented results indicate that the proposed scheme, combined with a suitable ML potential, could be employed to provide “ML/MM” energies with satisfactory precision. It also seems that with employing the embedding scheme to train a system-specific model, a considerable increase in precision can be expected. The architecture of the embedding scheme also allows taking advantage of other ML methods that aim at predicting electrostatic potential or electronic density, allowing reconciliation of the embedding model with the employed ML potential, resulting in lower computer costs and higher precision. We hope that this work will inspire interest in the development of ML embedding schemes and will enable the QM/MM community (for example, groups working on enzymatic catalysis) to take advantage of the rapidly growing toolbox of cheap and precise ML force fields.

## Data Availability

The Jupyter notebooks with the training (section 3.3) and prediction (section 3.4) procedures are available on GitHub (https://github.com/emedio/embedding). All the data needed to run the notebooks are available on Zenodo (10.5281/zenodo.7051785).

## Supporting Information Available

S1, molecules excluded from data set; S2, selection of reference atomic environments; S3, modified sparse GPR; S4, molecular dipolar polarizability from Thole model; S5, results with MBIS volumes; S6, calculation of electrostatic potential; S7, χ from MBIS partitioning; S8, prediction errors and standard deviations by element; S9, absolute embedding energy prediction errors; S10, GPR error estimate; S11, learning workflow; and S12, prediction workflow (PDF)
