# Supplementary material for: Electrostatic Embedding of Machine Learning Potentials
Source: J Chem Theory Comput. 2023 Feb 23;19(6):1888–97. doi: 10.1021/acs.jctc.2c00914 (PMC10061678; doi:10.1021/acs.jctc.2c00914)
Supplement: Supplementary file 1 — ct2c00914_si_001.pdf [file ct2c00914_si_001.pdf]

Electrostatic Embedding of Machine Learning Potentials: Supporting Information

Kirill Zinovjev*

Departament de Química Física, Universitat de València, 46100 Burjassot, Spain

E-mail: kirill.zinovjev@uv.es

S1 Molecules excluded from the dataset

Figure S1 shows four congeneric molecules excluded from the dataset. All have highly distorted geometries for the tertiary carbon atom in the three-membered ring, causing large prediction error for the atomic charge.

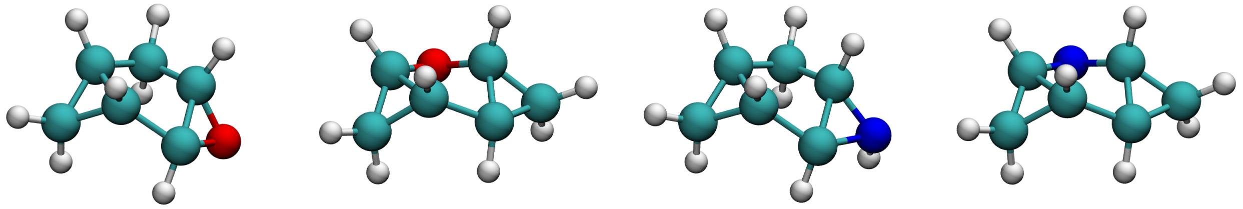

2155, $\Delta q_C = 0.98e$ 2161, $\Delta q_C = 0.70e$ 3999, $\Delta q_C = 0.53e$ 4117, $\Delta q_C = 0.46e$

Figure S1: Outliers excluded from the QM7 dataset.

S2 Selection of reference atomic environments

The reference environments were selected using Informative Vector Machine[1,2] approach: given a set of candidate datapoints, the process starts with a randomly picked datapoint and then adds the point which has the largest variance with the current model. The process

is repeated until the largest variance for any of the excluded datapoints falls below a certain threshold. An example of this process with a toy 1D model is presented on Figure S2.

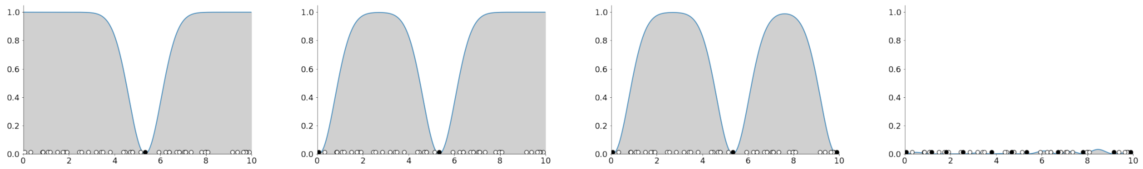

1 point   2 points   3 points   11 points

Figure S2: Informative Vector Machine.

Selecting 5% variance threshold with the SOAP features used in this work and a square polynomial kernel, the IVM process picks 445 reference environments out of 110650 atoms in the complete dataset. The number of atomic environments per element in QM7 and in the representative set are shown in Table S1.

Table S1: Number of atomic environments

	H	C	N	O	S	Total
QM7	61959	35761	6655	5978	297	110650
IVM (thr=0.05)	92	155	99	75	24	445

S3 Modified sparse GPR

As described in Deringer et al.,[3] sparse GPR approach aims to find the basis weights that minimize the prediction error on a large training set, while using only a limited number of basic atomic environments. The derivation is based on the assumption that the mean of the learned function is zero. If this is not the case, it is suggested that the mean is subtracted before learning and then added to the predictions. This essentially introduces an additional parameter to the model. To avoid that, here an alternative expression is used, which is based on the full GPR expression and provides the *values* of the learned function at the reference locations so that the prediction error over the full dataset is minimized. Additionally, a

constant shift is added to the predictions and the mean of the observations is constrained to zero. This is identical to subtracting the mean from the observations and adding it to the predictions. Moreover, learning the values instead of weights makes the free parameters physically meaningful, in a sense that they can be interpreted as an estimate of the physical observables corresponding to the basis locations based on the given training set. Therefore, they are much less dependent on a particular choice of features and kernel function compared to the GPR weights.

We start with the full GPR expression for N training observations and M basis locations ($M < N$):

$$\tilde{\mathbf{y}} = \mathbf{K}_{NM}\mathbf{c} = \mathbf{K}_{NM}\left[\mathbf{K}_{MM} + \mathbf{\Sigma}\right]^{-1}\mathbf{y}_{ref} \tag{S1}$$

Here $\tilde{\mathbf{y}}$ are N predictions, $\mathbf{c}$ are the GPR weights, $\mathbf{y}_{ref}$ are the observations for the basis locations, $\mathbf{K}_{NM}$ is the $N \times M$ kernel matrix for basis and reference locations, $\mathbf{K}_{MM}$ is the $M \times M$ symmetric kernel matrix for basis locations and $\mathbf{\Sigma}$ is the diagonal matrix of regularization weights. Now we modify the Eq. (S1) by introducing normalized observations $\mathbf{y}'_{ref}$ such that $\sum \mathbf{y}'_{ref} = 0$ and adding a constant shift y_0 to the predictions:

$$\tilde{\mathbf{y}} = \mathbf{A}\mathbf{y}'_{ref} + y_0\mathbf{1}_N \tag{S2}$$

where we introduced $\mathbf{A} = \left[\mathbf{K}_{MM} + \mathbf{\Sigma}\right]^{-1}\mathbf{K}_{MN}$ for clarity and $\mathbf{1}_N$ is an N-vector of ones.

Now, the values $\mathbf{y}'_{ref}$ can be optimized by minimizing the following loss function:

$$\mathscr{L} = \sum_i \left(\tilde{y}_i - y_i\right)^2 + \lambda \sum_i y'_{ref_i} = \left(\tilde{\mathbf{y}} - \mathbf{y}\right)^T\left(\tilde{\mathbf{y}} - \mathbf{y}\right) + \lambda \sum \mathbf{y}'_{ref} \tag{S3}$$

Where $\mathbf{y}$ are the training observations and λ is a Lagrange multiplier. By differentiating Eq.

(S3) w.r.t. $\mathbf{y}'_{ref}$ and y_0 we get the following set of equations:

$$\begin{cases} \mathbf{A}\left(\tilde{\mathbf{y}} - \mathbf{y}\right) + \lambda \mathbf{1}_N = \mathbf{0} \\ \sum \mathbf{y}'_{ref} = 0 \end{cases} \tag{S4}$$

solving which gives:

$$\begin{aligned} y_0 &= \frac{\mathbf{1}_M^T \mathbf{B} \mathbf{y}}{\mathbf{1}_M^T \mathbf{B} \mathbf{1}_N} \\ \mathbf{y}'_{ref} &= \mathbf{B}\mathbf{y} - y_0 \mathbf{B} \mathbf{1}_N \end{aligned} \tag{S5}$$

where $\mathbf{B} = \left(\mathbf{A}\mathbf{A}^T\right)^{-1} \mathbf{A}$ is the Moore-Penrose pseudoinverse of $\mathbf{A}^T$. The observations for the basis locations that serve as the free parameters of the model are then:

$$\mathbf{y}_{ref} = \mathbf{y}'_{ref} + y_0 \mathbf{1}_M \tag{S6}$$

S4 Molecular dipolar polarizability from Thole model

The molecular dipolar polarizability tensor $\boldsymbol{\alpha}_{mol}$ provides the induced molecular dipole $\boldsymbol{\mu}_{ind}$ in response to the uniform external electric field $\mathbf{E}$ through the following expression:

$$\boldsymbol{\mu}_{ind} = \boldsymbol{\alpha}_{mol} \mathbf{E} \tag{S7}$$

In the induction model presented in this work, the induced dipole can be calculated as a sum of induced atomic dipoles obtained with the Thole model, $\boldsymbol{\mu}_{ind} = \sum_i \boldsymbol{\mu}_i$. Then, $\boldsymbol{\alpha}_{mol}$ is

obtained as follows:

$$\begin{pmatrix} \boldsymbol{\alpha}_1^{-1} & -\mathbf{T}_{12} & \cdots & -\mathbf{T}_{1N} \\ -\mathbf{T}_{21} & \boldsymbol{\alpha}_2^{-1} & \cdots & -\mathbf{T}_{2N} \\ \vdots & \vdots & \ddots & \vdots \\ -\mathbf{T}_{N1} & -\mathbf{T}_{N2} & \ldots & \boldsymbol{\alpha}_N^{-1} \end{pmatrix} \begin{pmatrix} \boldsymbol{\mu}_1 \\ \boldsymbol{\mu}_2 \\ \vdots \\ \boldsymbol{\mu}_N \end{pmatrix} = \mathbf{A} \begin{pmatrix} \boldsymbol{\mu}_1 \\ \boldsymbol{\mu}_2 \\ \vdots \\ \boldsymbol{\mu}_N \end{pmatrix} = \begin{pmatrix} \mathbf{E} \\ \mathbf{E} \\ \vdots \\ \mathbf{E} \end{pmatrix} \tag{S8}$$

$$\begin{pmatrix} \boldsymbol{\mu}_1 \\ \boldsymbol{\mu}_2 \\ \vdots \\ \boldsymbol{\mu}_N \end{pmatrix} = \mathbf{A}^{-1} \begin{pmatrix} \mathbf{E} \\ \mathbf{E} \\ \vdots \\ \mathbf{E} \end{pmatrix} = \mathbf{B} \begin{pmatrix} \mathbf{E} \\ \mathbf{E} \\ \vdots \\ \mathbf{E} \end{pmatrix} = \begin{pmatrix} \mathbf{b}_{11} & \mathbf{b}_{12} & \cdots & \mathbf{b}_{1N} \\ \mathbf{b}_{21} & \mathbf{b}_{22} & \cdots & \mathbf{b}_{2N} \\ \vdots & \vdots & \ddots & \vdots \\ \mathbf{b}_{N1} & \mathbf{b}_{N2} & \ldots & \mathbf{b}_{NN} \end{pmatrix} \begin{pmatrix} \mathbf{E} \\ \mathbf{E} \\ \vdots \\ \mathbf{E} \end{pmatrix} \tag{S9}$$

$$\boldsymbol{\mu}_{ind} = \sum_i \boldsymbol{\mu}_i = \left(\sum_{ij} \mathbf{b}_{ij} \right) \mathbf{E} \Rightarrow \boldsymbol{\alpha}_{mol} = \sum_{ij} \mathbf{b}_{ij} \tag{S10}$$

where $\mathbf{B} = \mathbf{A}^{-1}$ and $\mathbf{b}_{ij}$ are 3x3 blocks of $\mathbf{B}$.

S5 Results with MBIS volumes

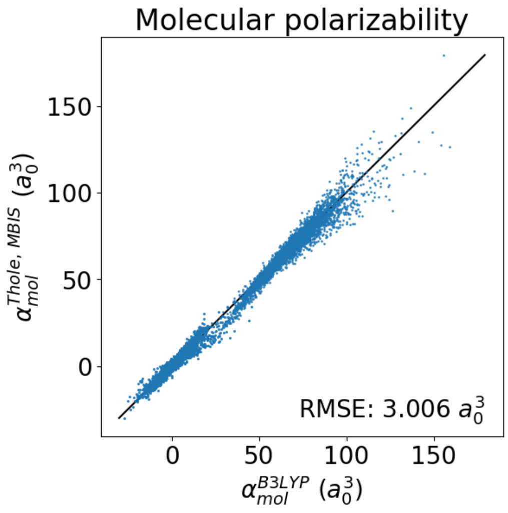

(a)

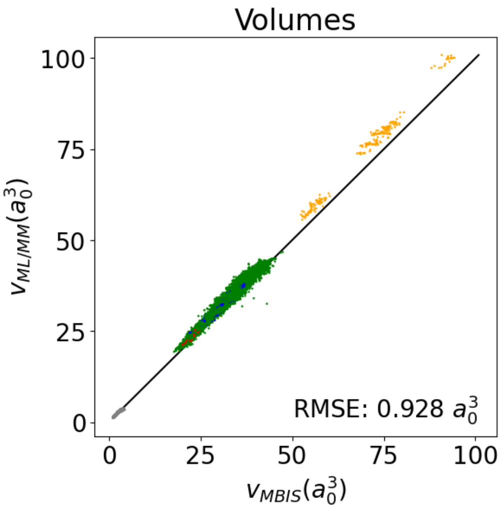

(b)

Figure S3: a) Predicted molecular polarizability components based on MBIS volumes vs B3LYP values; b) Predicted volumes vs MBIS ones.

S6 Calculation of electrostatic potential

The electrostatic potentials created by the QM subsystem at the MM atomic positions were calculated using `orca_vpot`. The GFN2-xTB reference calculations were also done through ORCA interface, which does not support generation of density files, so for GFN2-xTB the potentials were not calculated. In the case of AM1 and PM3, it seems to be a bug in either `orca` or `orca_vpot`, resulting in huge errors for the seliempirical electrostatic potential values compared to the DFT ones (RMSE $\approx 10\ E_h/e$), so these were not used for discussion. The bug is reported (`https://orcaforum.kofo.mpg.de/viewtopic.php?f=11&t=9451`).

S7 χ from MBIS partitioning

While electronegativities are not directly available from QM reference data and are obtained by sparse GPR fitting to MBIS charges, once the scaling factor a_{QEq} is known, it is possible to derive χ from the MBIS atomic charges by rearranging Eq. 16:

$$\begin{pmatrix} \chi_1^{MBIS} \\ \chi_2^{MBIS} \\ \vdots \\ \chi_N^{MBIS} \end{pmatrix} = \begin{pmatrix} J_1 & E_{12}^{int} & \cdots & E_{1N}^{int} \\ E_{21}^{int} & J_2 & \cdots & E_{2N}^{int} \\ \vdots & \vdots & \ddots & \vdots \\ E_{N1}^{int} & E_{N2}^{int} & \dots & J_N \end{pmatrix} \begin{pmatrix} -q_1^{MBIS} \\ -q_2^{MBIS} \\ \vdots \\ -q_N^{MBIS} \end{pmatrix} \tag{S11}$$

The obtained electronegativities provide exactly the MBIS charges when used in QEq with the same parameters (valence widths and a_{QEq}). "Average" electronegativities ($\langle \chi_{MBIS} \rangle$) used in Table 3 are obtained by averaging χ^{MBIS} values for each atom over the dataset.

S8 Prediction errors and standard deviations by element

Table S2: Absolute / relative prediction errors for valence widths and charges split by chemical element. In parenthesis the standard deviations are given.

Element	Valence width (Å)	Charge (e)
H	0.003 / 0.300 (0.011)	0.014 / 0.163 (0.085)
C	0.003 / 0.209 (0.014)	0.027 / 0.089 (0.301)
N	0.002 / 0.198 (0.009)	0.027 / 0.119 (0.228)
O	0.001 / 0.184 (0.005)	0.022 / 0.161 (0.135)
S	0.003 / 0.209 (0.013)	0.025 / 0.040 (0.621)

S9 Absolute embedding energy prediction errors

Table S3: Absolute energy prediction RMSE (kcal/mol)

Method	E_{Full}	E_{Static}	$E_{Induced}$
PBE0/cc-pVTZ	0.522	0.635	0.123
B3LYP/cc-pVDZ	4.312	2.263	2.081
BLYP/6-31G*	4.884	2.728	2.189
ML/MM q_{MBIS}	5.380	5.316	0.561
ML/MM MBIS	5.354	5.333	0.549
ML/MM $\langle \chi_{MBIS} \rangle$	5.303	5.286	0.562
ML/MM $\langle q_{MBIS} \rangle$	5.939	5.918	0.561
ML/MM	3.263	3.178	0.567
GFN2-xTB	34.583	-	-
AM1	68.266	-	-
PM3	77.713	-	-

S10 GPR error estimate

To estimate the error of embedding energy prediction associated specifically to the GPR approximation of atomic properties, below the ML/MM embedding energies are compared to the ones obtained with exact MBIS values (ML/MM MBIS in Table 3). The normalised RMSE is 1.75 kcal/mol, comparable to the error between ML/MM MBIS and reference DFT calculations (1.49 kcal/mol).

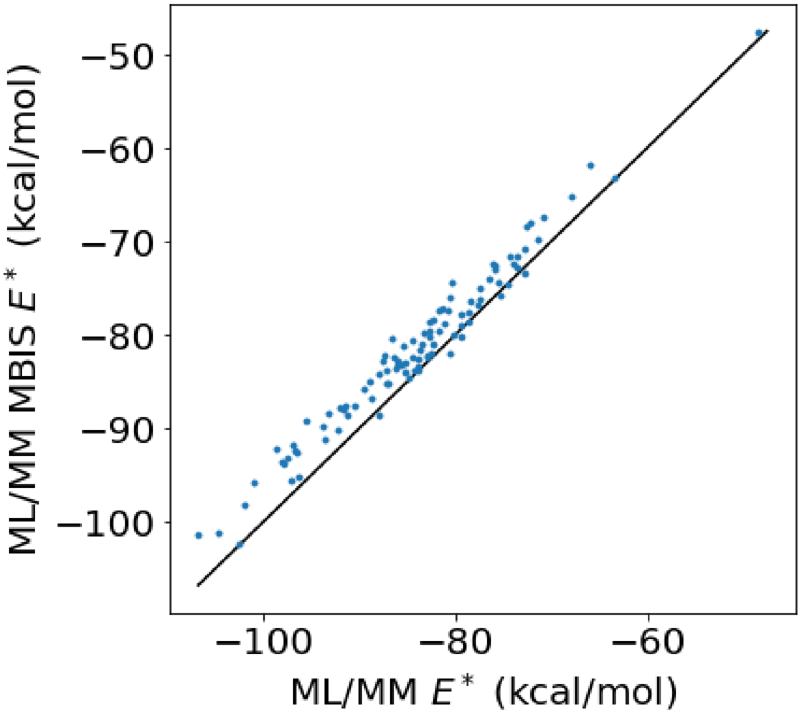

S11 Learning workflow

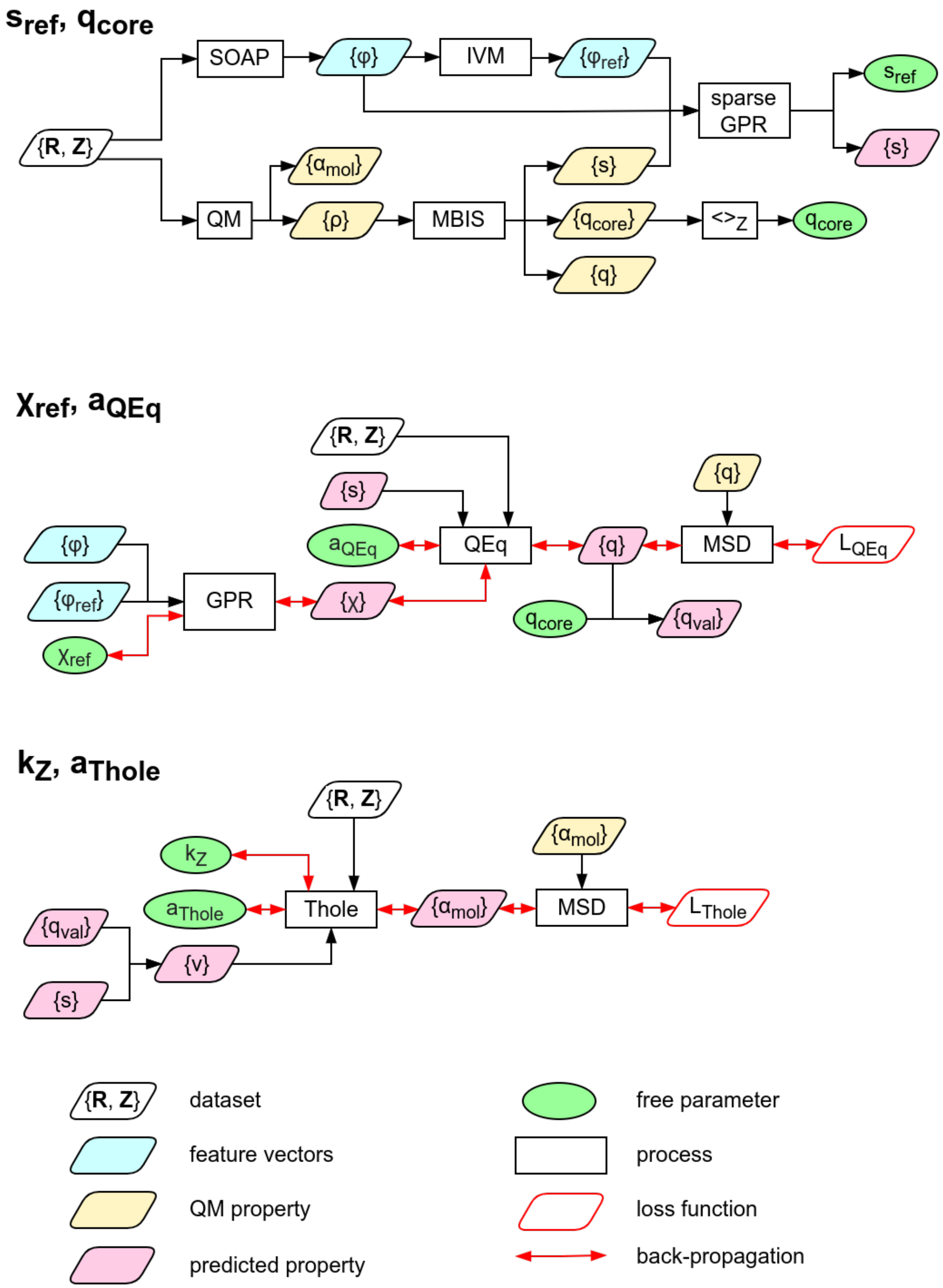

Figure S4: Learning workflow.

S12 Prediction workflow

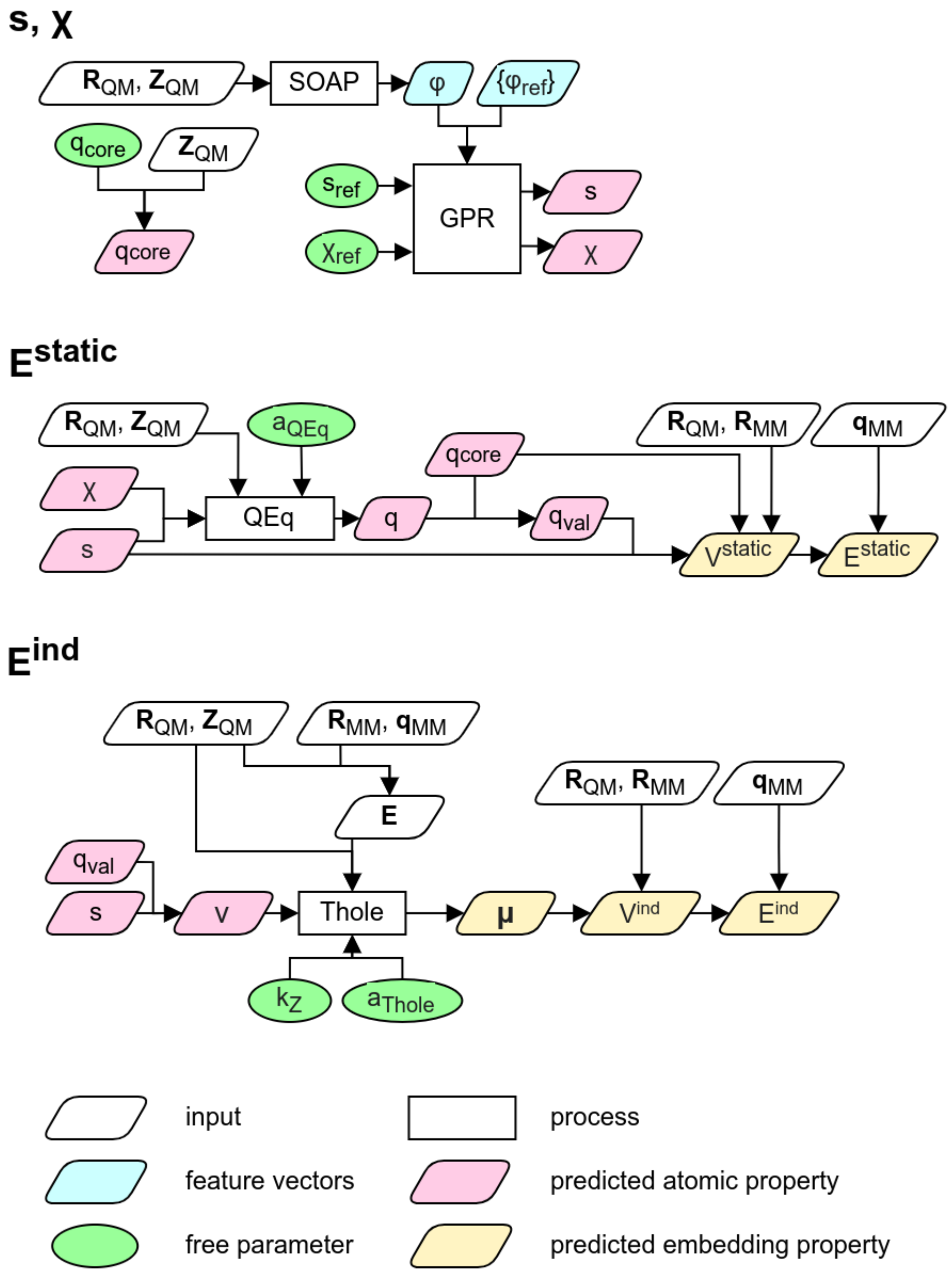

Figure S5: Prediction workflow.

References

(1) Rasmussen, C. E.; Williams, C. K. I. *Gaussian processes for machine learning*; Adaptive computation and machine learning; MIT Press: Cambridge, Mass, 2006; OCLC: ocm61285753.

(2) Herbrich, R.; Lawrence, N. D.; Seeger, M. Fast Sparse Gaussian Process Methods: The Informative Vector Machine. 8.

(3) Deringer, V. L.; Bartók, A. P.; Bernstein, N.; Wilkins, D. M.; Ceriotti, M.; Csányi, G. Gaussian Process Regression for Materials and Molecules. *Chem. Rev.* **2021**, *121*, 10073–10141.
